# Waterpipe Tobacco Smoking and Gastric Cancer Risk among Vietnamese Men

**DOI:** 10.1371/journal.pone.0165587

**Published:** 2016-11-01

**Authors:** Hang Thi Minh Lai, Chihaya Koriyama, Shinkan Tokudome, Hoc Hieu Tran, Long Thanh Tran, Athira Nandakumar, Suminori Akiba, Ngoan Tran Le

**Affiliations:** 1 Department of Epidemiology and Preventive Medicine, Kagoshima University Graduate School of Medical and Dental Sciences, Kagoshima, Japan; 2 National Institute of Health and Nutrition, Tokyo, Japan; 3 Department of Surgery, Hanoi Medical University, Hanoi, Vietnam; 4 Department of Occupational Health, Hanoi Medical University, Hanoi, Vietnam; Virginia Commonwealth University, UNITED STATES

## Abstract

**Background:**

The association of waterpipe tobacco (WPT) smoking with gastric cancer (GC) risk was suggested.

**Methods:**

A hospital-based case-control study was conducted to examine the association of WPT with GC risk among Vietnamese men, in Hanoi city, during the period of 2003–2011. Newly-diagnosed GC cases (n = 454) and control patients (n = 628) were matched by age (+/- 5 years) and the year of hospitalization. Information on smoking and alcohol drinking habits and diet including salty food intake and fruits/vegetables consumption were obtained by the interview. Maximum likelihood estimates of odds ratios (ORs) and corresponding 95% confidence intervals (Cis) were obtained using conditional logistic regression models.

**Results:**

The group with the highest consumption of citrus fruits showed a significantly low GC risk (OR = 0.6, 95%CI = 0.4–0.8, P for trend = 0.002). However, there was no association of raw vegetable consumption with GC risk. Referring to never smokers, GC risk was significantly higher in current WPT smokers (OR = 1.8, 95%CI = 1.3–2.4), and it was more evident in exclusively WPT smokers (OR = 2.7, 95%CI = 1.2–6.5). GC risk tended to be higher with daily frequency and longer duration of WPT smoking but these trends were not statistically significant (P for trend: 0.144 and 0.154, respectively). GC risk of those who started smoking WPT before the age of 25 was also significantly high (OR = 3.7, 95%CI = 1.2–11.3). Neither cigarette smoking nor alcohol drinking was related to GC risk.

**Conclusion:**

The present findings revealed that WPT smoking was positively associated with GC risk in Vietnamese men.

## Introduction

Although an involvement of cigarette smoking in the development of gastric cancer (GC) has been reported in several studies [1–5], evidence of the association between waterpipe tobacco (WPT) and GC risk is limited. A case-control study conducted in Northeast Iran did not find a significant association between GC risk and hookah, an Arabian type of WPT [6], because of the small number of hookah smokers. However, a recent cohort study reported that GC risk significantly increased to three-fold among hookah smokers in a specific cohort, *Helicobacter pylori (H*. *pylori)*-infected healthy subjects in Northwest Iran [7].

Arabian waterpipe, also known as hookah, shisha, or narghile, is a centuries-old device to smoke tobacco. Its use has recently grown among young populations in Western countries due to the common belief that WPT is less harmful than cigarette. To smoke WPT, tobacco is heated by burning charcoal to produce smoke that passes through a column of water before being inhaled [8]. One typical session of hookah smoking lasts around 45–60 minutes [9]. Several studies have shown that hookah smoke contains a variety of carcinogenic and toxic substances such as polycyclic aromatic hydrocarbons (PAHs), tobacco-specific nitrosamines, carbonyls, carbon monoxide (CO), and heavy metals [10–13].

**Fig 1:**
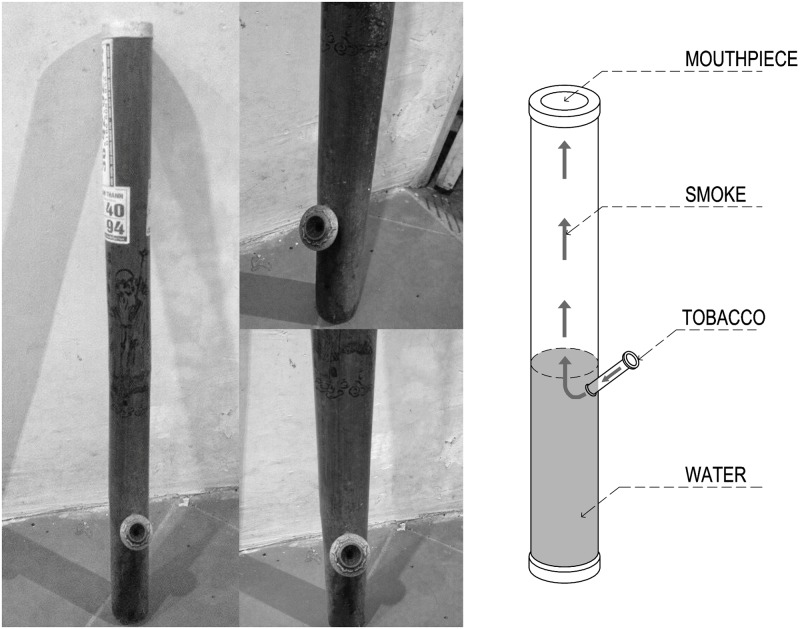
Vietnamese/Chinese waterpipe.

According to the National Health Survey in Vietnam, the prevalence of male smokers was 51.2%, and most of them smoked cigarette only (69.1%), followed by Vietnamese WPT only (23.2%) and both products (7.7%) in 2001–2002 [14]. Vietnamese waterpipe is made of bamboo (Fig 1) and its structure and the direction for use are quite similar to one used in China [15]. Tobacco leaf used in Vietnamese WP smoking is a plant called Nicotiana rustica, which has a higher level of nicotine (9%) than that of cigarette (1–3%). WP tobacco is prepared from the leaves which are shredded and sundried or sometimes dried in large wood burning kilns. The smoking method of Vietnamese/Chinese WPT is similar with that of the Arabian WPT whereby smoke passes through water before being inhaled [16]. Unlike the Arabian WPT, Vietnamese/Chinese WPT does not require charcoal, and each smoking session is generally short, usually lasting less than 5 minutes. Although charcoal, which was suspected as a main source of CO and PAHs [17], is not used in Vietnamese/Chinese WPT, She *et al*. [16] observed that the exhaled CO level among Chinese WPT smokers was significantly higher than those of non-smokers and even cigarette smokers. This finding suggests a possibility that the smoke of Vietnamese/Chinese WPT also contains high levels of CO and PAHs despite charcoal not being used. Similarly to Arabian WPT, other carcinogens such as tobacco-specific nitrosamines and heavy metals most likely exist in the smoke of Vietnamese/Chinese WPT.

To our knowledge, there have been no studies examining the association between Vietnamese WPT smoking and GC risk. In this study, we aimed to clarify the association of Vietnamese WPT smoking with GC risk among men, since the proportion of female WPT smokers is too low in Vietnam (0.1%) [18].

## Materials and Methods

### Selection of cases and controls

A hospital-based case-control study for GC was performed in the Hanoi city, Vietnam, during the following three study periods; first study: February 2003—August 2006, second study: September 2006—November 2007, and third study: November 2010—April 2011. All study subjects were recruited from three major hospitals in Hanoi; Hanoi Cancer Hospital, Viet Duc Surgery Hospital, and Bach Mai General Hospital. Cases were Vietnamese male patients diagnosed as primary GC histopathologically, and 495 GC cases were recruited. Controls were also Vietnamese male patients hospitalized in the same hospitals and during the same study period, and 692 hospital patients without history of any cancer were recruited. The top-five diseases of control patients were urinary stone (15.7%), gall stone (14.5%), trauma (12.7%), benign prostatic hyperplasia (10.3%), and inguinal hernia (5.9%).

**Fig 2:**
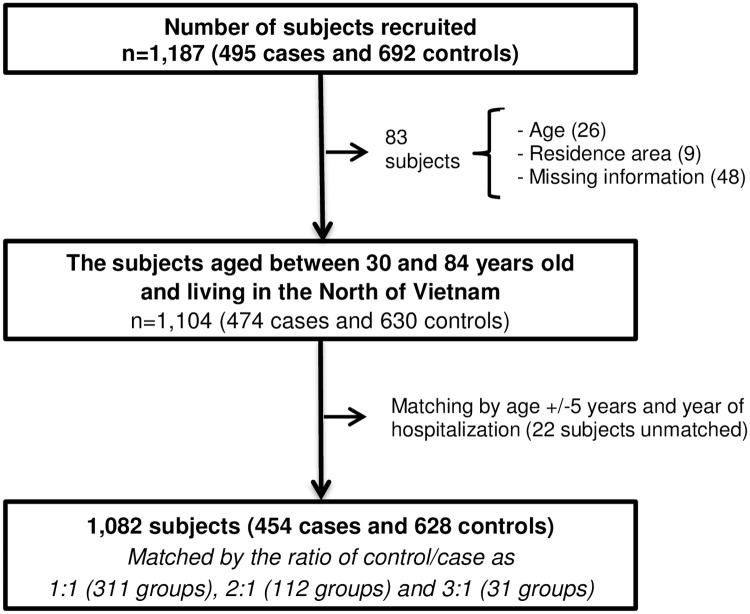
Process of subject recruitment.

Subjects aged 30–84 and living in the North Vietnam were included in the present study. Patients under the age of 30 were excluded as GC risk is low [19] and the exposure period of smoking might be too short for GC development. Patients over the age of 84 were also excluded as clinical diagnosis and the information on exposure and confounding factors for elderly are generally unreliable [20]. In summary, the exclusion criteria and the corresponding number of the excluded subjects were as follows (Fig 2): i) aged under 30 or over age 84 (8 cases and 18 controls), ii) residents outside of the North Vietnam (2 cases and 7 controls), and iii) missing information on smoking (11 cases and 37 controls). Furthermore, 20 cases and 2 controls could not be matched by age and the year of hospitalization. Thus, 41 cases and 64 controls were excluded, and 454 (91.7%) cases and 628 (90.8%) controls were used for the present analysis. Control(s) were re-matched with a case for age (+/- 5 years) and the calendar year of hospitalization, and the numbers of matched control for one case were one for 311 groups, two for 112 groups, and three for 31 groups.

### Questionnaire and data collection

Face to face interviews were conducted using a structured questionnaire by trained interviewers. Socio-demographic factors, cancer history for both patients and his family members, smoking and alcohol drinking habits and other lifestyles including diet were contained in the questionnaire. Information on the location of tumors was obtained from the medical records.

Regarding the socio-demographic factors, age at the time of interview, place of residence, education, and occupation were asked. The refrigerator use, which is considered as one of protective factors for GC, was also asked as one of indicators for the socio-economic status. A previous review article on epidemiology of GC reported that refrigerators improved the storage of food, thereby led to decrease in intake of preserved foods which generally have high salt content [21]. Additionally, an ecological study conducted in Korea showed a negative association between refrigerator use and GC mortality [22].

For tobacco smoking, all subjects were classified into three categories: never smokers, ex-smokers, and current smokers. According to the U.S. Centers for Disease Control, never smokers were defined as those who never smoked or smoked less than 100 cigarettes/WPTs in their lifetime [23]. Subjects who had smoked cigarette/WPT regularly at least for one year was defined as smokers. The definition of ex-smokers were persons who had smoked in the past but quit at least one year before the onset/symptom(s) of disease which was the reason of hospitalization. Other smokers were treated as current smokers. Smokers were asked about the types of tobacco (cigarette, WPT, or both types) they smoke, frequency and average duration of smoking, and age when they start smoking. The use of other tobacco products was not investigated in the present study because the proportion of male smokers is negligible in Vietnam. According to Global Adult Tobacco Survey in Vietnam 2010, the proportion of men who used smokeless tobacco and other tobacco products including cigar and pipe was 0.3% and 0.2%, respectively [18].

Information on the frequency of alcohol drinking, salted-processed meats and dried fish, citrus fruits (lemon, orange, grapefruit, tangerine, and pomelo) and raw vegetables was also obtained.

### Statistical analysis

For the statistical analysis, we classified the resident area as Hanoi, Red River Delta (excluding Hanoi) and others (North East, North West and North Central Coast) since lifestyles and socio-economic status might be different among them. Based on schooling years, we categorized the educational level as follows: ≤ 6 years (equal to primary school or lower level), 7–9 years (secondary school), 10–12 years (high school), and more than 12 years (university or higher). Occupation was divided into six groups as retiree, farmer, factory worker, office worker, free labor, and others.

All WPT smokers in our study used the Vietnamese-type WP. Based on previous reports [2, 24, 25] and the distribution of the study subjects by each exposure factor of WPT smoking, exposure factors were analyzed using the following categories: the daily frequency (< 10 and ≥ 10 per day), duration of smoking (< 20, 20–29, and ≥ 30 years) and age at start smoking (< 25 and ≥ 25 years old). Furthermore, cumulative frequency (CF) of WPT smoking was calculated by multiplying the average daily frequency of WPTs, 365 days, and the duration of smoking (years) [26]. We divided this index into three groups: < 100,000, ≥ 100,000, and “not determined” because of missing information either daily frequency or duration of smoking. Never-smoker group was always used as reference in the statistical analyses for these factors.

Frequency of alcohol drinking was divided into three levels: never (never or 2–3 days per year), sometimes (less than 3 days per week), and frequent (≥ 3 days per week). For salty foods, we categorized the frequency of salted-processed meats and dried fish intake into three groups: never or rarely (never use or 1–2 times per year), monthly (at least 1–2 times per month), and daily or weekly (at least 1–2 times per week).

The IARC working group concluded that a higher intake of fruits and vegetables “probably” and “possibly” could reduce the risk of GC, respectively [27]. Citrus fruits are rich in vitamin C that could influence cancer development by scavenging reactive oxygen species, protecting mucosal tissues from the damaging effects of oxidative stress, and inhibiting nitrosamine formation in the stomach [28]. In the present study, thus, the consumption of raw vegetables and citrus fruits was considered as confounding factors. Average daily consumption of raw vegetables was converted from the data of weekly/monthly consumption. For citrus fruits, cumulative daily consumption was also calculated based on the information of weekly or monthly consumption of lemon, orange, grapefruit, tangerine, and pomelo. Calculated daily consumptions of raw vegetables and citrus fruits were divided into tertiles according to their distributions in control patients.

Although *H*. *pylori* infection is a well-known established risk factor for gastric cancer [21], the IARC working group concluded that “*H*. *pylori* is of little or no relevance with regard to potential confounding of the association between (cigarette) smoking and stomach cancer [24]. Therefore, the information on *H*. *pylori* infection was not taken into account in the analysis.

A conditional logistic regression model was applied to obtain the maximum likelihood estimates of Ors and corresponding 95% confidence intervals (Cis) of GC risk. For multivariable analysis, we adjusted for the effects of potential confounding factors including age, education, residential area, and frequency of salty foods, citrus fruits and raw vegetables intake. Variables of education and dietary intake were treated as an ordinal variable. A trend test was conducted using ordinal variables after categorization. P values for homogeneity were estimated using the likelihood ratio test. All P values were two-sided.

### Ethical approval

This study was approved by the Ministry of Health and Ministry of Education in Vietnam and the Ethics Committee of Kagoshima University Graduate School of Medical and Dental Sciences in Japan.

We obtained verbal informed consents from all participants. According to the guideline for epidemiological study in Vietnam and Japan in 2002, a written informed consent was not required for observational study based on questionnaire survey. The informed consents were implied if the participants completed the questionnaire.

## Results

The proportion of the subjects recruited in each study period was 23%, 54%, and 23% for the study 1, study 2, and study 3, respectively.

### Socio-demographic factors

The means (SD) of age for GC cases and control patients were 56.7 (11.1) and 56.7 (11.3) years, respectively. Most of the subjects lived in Red River Delta (68.9%, of which 23.7% in Hanoi), 15.4% in northern midland and mountain area (North West and North East) and 15.7% in North Central Coast.

The education level showed a significant inverse association with GC risk (P for trend = 0.003), and farmers showed the highest GC risk (OR = 2.0, 95%CI = 1.3–2.9) in comparison with retiree (Table 1). Relatively large number of “unknown” subjects for occupation was due to the absence of this item in the questionnaire of the study 1. The use of refrigerator significantly lowered GC risk (OR = 0.6, 95%CI = 0.5–0.8). No association was observed between family history of cancer and GC risk in this study.

**Table 1:** The effects of socioeconomic status and other factors on the risk of gastric cancer.

Variables	Control		Gastric cancer		OR (95%CI)^b^	P-value^c^
	n	%	n	%		
**Total**	628	100	454	100		
**Resident area**						0.361
Ha Noi	109	17.4	68	15.0	1.0	
Red River Delta	334	53.2	236	52.0	1.2 (0.9–1.7)	
Others	185	29.5	150	33.0	1.3 (0.9–1.9)	
**Education (years)**						0.034
<6	57	9.1	56	12.3	1.0	P for trend = 0.003^b^
6–9	282	44.9	223	49.1	0.7 (0.5–1.1)	
10–12	180	28.7	116	25.6	0.6 (0.4–1.0)	
≥12	106	16.9	57	12.6	0.5 (0.3–0.8)	
Unknown	3	0.5	2	0.4	0.5 (0.1–3.3)	
**Occupation**						0.006
Retiree	166	26.4	83	18.3	1.0	
Farmer	138	22.0	138	30.4	2.0 (1.3–2.9)	
Factory worker	31	4.9	20	4.4	1.4 (0.7–2.6)	
Office worker	33	5.3	15	3.3	0.8 (0.4–1.7)	
Free labor and others	75	11.9	43	9.5	1.1 (0.7–1.9)	
Unknown	185	29.5	155	34.1	1.8 (0.8–3.9)	
**Refrigerator**						0.002
No	248	39.5	219	48.2	1.0	
Yes	339	54.0	199	43.8	0.6 (0.5–0.8)	
Unknown	41	6.5	36	7.9	0.9 (0.5–1.4)	
**Frequency of salted processed meats and dried fish intake**						0.070
Never or rarely	210	33.4	143	31.5	1.0	P for trend = 0.105^b^
Monthly	338	53.8	220	48.5	0.9 (0.7–1.2)	
Daily/weekly	78	12.4	90	19.8	1.5 (1.0–2.2)	
Unknown	2	0.3	1	0.2	0.8 (0.1–9.6)	
**Frequency of citrus fruits consumption**^a^						0.007
T1	212	33.8	187	41.2	1.0	P for trend = 0.002^b^
T2	206	32.8	161	35.5	0.9 (0.7–1.3)	
T3	208	33.1	104	22.9	0.6 (0.4–0.8)	
Unknown	2	0.3	2	0.4	1.1 (0.1–7.8)	
**Frequency of raw vegetables consumption**^a^						0.204
T1	257	40.9	176	38.8	1.0	P for trend = 0.756^b^
T2	173	27.6	151	33.3	1.2 (0.9–1.6)	
T3	198	31.5	126	27.8	0.9 (0.7–1.3)	
Unknown	0	0.0	1	0.2	-	
**Family history of cancer**						0.134
No	578	92.0	412	90.8	1.0	
Gastric cancer	9	1.4	12	2.6	2.3 (0.9–5.5)	
Other cancers	24	3.8	21	4.6	1.2 (0.7–2.3)	
Unknown cancer	0	0.0	1	0.2	-	
Unknown	17	2.7	8	1.8	0.7 (0.3–1.7)	

Abbreviation: OR, odds ratio; 95%CI, 95% confidence interval

^a^T1-T3: Tertile of frequency of citrus fruits consumptions (T1 <0.17, T2 <0.6, T3 ≥0.6) and frequency of raw vegetables consumptions (T1 = 0, T2<0.08, T3≥0.08)

^b^OR and corresponding 95%CI and p-value were obtained by conditional logistic regression models. P for trend was estimated excluding unknown group.

^c^P values for homogeneity were estimated using likelihood ratio test.

### Dietary factors

The group with the highest consumption of citrus fruits showed a significantly low GC risk (OR = 0.6, 95%CI = 0.4–0.8, P for trend = 0.002). However, there was no association of raw vegetable consumption with GC risk. Those who consumed salted processed meats and dried fish at least 1–2 times per week showed a higher risk of GC (OR = 1.5, 95%CI = 1.0–2.2).

### Selection of confounding factors

There were strong correlations among education, occupation and refrigerator use (P values <0.001). Because of the small number of missing information, education was taken account as one of confounding variables for further analyses in addition to age, resident area, salted-processed meats and dried fish, citrus fruits and raw vegetable consumption.

### Smoking and alcohol drinking

The WPT smoking was positively associated with GC risk (Table 2). GC risk in current WPT smokers was significantly high (OR = 1.8, 95%CI = 1.3–2.4), and ex-smokers also showed an increase trend (OR = 1.5, 95%CI = 1.0–2.4). On the other hand, cigarette smoking and alcohol drinking were not related to GC risk.

**Table 2:** The effect of cigarette and waterpipe tobacco smoking and alcohol drinking on the risk of gastric cancer.

Variables	Control		Gastric cancer		OR (95%CI)^a^	P-value^b^
	n	%	n	%		
**Total**	**628**	**100**	**454**	**100**		
**Cigarette smoking**						0.547
Never	238	37.9	168	37.0	1.0	
Ex-smoker	117	18.6	94	20.7	1.2 (0.9–1.7)	
Current smoker	273	43.5	192	42.3	1.1 (0.8–1.4)	
**Waterpipe tobacco smoking**						<0.001
Never	388	61.8	219	48.2	1.0	
Ex-smoker	69	11.0	56	12.3	1.5 (1.0–2.4)	
Current smoker	171	27.2	179	39.4	1.8 (1.3–2.4)	
**Alcohol drinking**						0.605
Never	194	30.9	121	26.7	1.0	
Some times	222	35.4	175	38.6	1.0 (0.7–1.4)	
Frequent	210	33.4	156	34.4	1.2 (0.8–1.6)	
Unknown	2	0.3	2	0.4	1.8 (0.2–20.9)	

Abbreviation: OR, odds ratio; 95%CI, 95% confidence interval

^a^OR and corresponding 95%CI were obtained by conditional logistic regression models with adjusting for the effects of age, education, resident area, intake of salted processed meats and dried fish, and consumption of citrus fruits and raw vegetables.

^b^P values for homogeneity were estimated using likelihood ratio test.

For WPT smoking, GC cases had a higher frequency per day, longer duration, and earlier start of smoking than those of controls (Table 3). There was no significant difference in the number of cigarettes per day between them.

**Table 3:** Characteristics of study subjects regarding smoking status.

Variable		Control	Gastric cancer	Total
		n = 628	n = 454	n = 1082
**Current cigarette smoking only (%)**		**25.3**	**21.8**	**23.8**
No. cigarettes per day	Mean (SD)	10.6 (7.1)	9.1 (6.9)	10.1 (7.1)
	Median	10	7	10
**Both current cigarette and ever WPT smoking (%)**		**18.2**	**20.5**	**19.1**
No. cigarettes per day	Mean (SD)	8.3 (6.8)	8.7 (6.1)	8.5 (6.5)
	Median	5.5	8	6
**Both current WPT and ever cigarette smoking (%)**		**18.0**	**21.6**	**19.5**
No. WPTs per day	Mean (SD)	8.4 (7.9)	8.9 (7.2)	8.6 (7.6)
	Median	5	6.3	5.8
**Current WPT smoking only (%)**		**9.2**	**17.8**	**12.9**
No. WPTs per day	Mean (SD)	9.2 (6.2)	11.3 (7.8)	10.4 (7.3)
	Median	8.5	10	10
Years of smoking	Mean (SD)	29.4 (13.3)	34.3 (12.6)	30.5 (12.8)
Age at starting to smoke	Mean (SD)	29.4 (14.3)	26.7 (11.0)	26.8 (11.3)

Abbreviation: SD, standard deviation

We further examined the association of WPT smoking with GC risk using exclusively WPT smokers (Table 4). The high GC risk was more evident among current WPT smokers (OR = 2.7, 95%CI = 1.2–6.5). Those who smoked WPT 10 or more times per day also showed a significantly high GC risk (OR = 2.9, 95%CI = 1.0–8.3). The daily frequency and longer duration of WPT smoking tended to be higher GC risk although there was no statistical significance. Early start of smoking was also related to the higher risk of GC. Those who started smoking before the age of 25 showed a high risk of GC (OR = 3.7, 95%CI = 1.2–11.3) in comparison with never smokers.

**Table 4:** Waterpipe tobacco smoking and gastric cancer risk, excluding cigarette smokers.

Variables for waterpipe tobacco smoking	Control		Gastric cancer		OR (95%CI)^a^	P-value^d^
	n	%	n	%		
**Total**	105	100	88	100		
**Waterpipe tobacco only**						0.055
Never	71	67.6	45	51.1	1.0	P for trend = 0.024^b^
Ex-smoker	9	8.6	8	9.1	1.2 (0.3–4.1)	
Current smoker	25	23.8	35	39.8	2.7 (1.2–6.5)	
**Frequency (per day)**						0.317
Never	71	67.6	45	51.1	1.0	P for trend = 0.144^c^
<10	11	10.5	13	14.8	2.0 (0.7–6.0)	
10 or more	13	12.4	19	21.6	2.9 (1.0–8.3)	
Unknown	1	1.0	3	3.4	7.9 (0.6–103.9)	
**Cumulative frequency**						0.554
Never	71	67.6	45	51.1	1.0	P for trend = 0.284^c^
<100,000	10	9.5	11	12.5	2.1 (0.7–6.6)	
100,000 or more	12	11.4	18	20.5	3.0 (1.0–9.0)	
Unknown	3	2.9	6	6.8	3.9 (0.8–20.5)	
**Smoking duration (years)**						0.515
Never	71	67.6	45	51.1	1.0	P for trend = 0.154^b^
<20	7	6.7	7	8.0	1.3 (0.4–4.6)	
20–29	6	5.7	9	10.2	2.4 (0.6–10.2)	
30 or more	18	17.1	22	25.0	2.7 (0.9–8.0)	
Unknown	3	2.9	5	5.7	2.9 (0.5–15.8)	
**Starting age for waterpipe tobacco smoking (years)**						0.103
<25	10	9.5	18	20.5	3.7 (1.2–11.3)	P for trend = 0.249^b^
25 or more	18	17.1	20	22.7	1.9 (0.8–4.7)	
Never	71	67.6	45	51.1	1.0	
Unknown	6	5.7	5	5.7	1.5 (0.4–6.2)	

Abbreviation: OR, odds ratio; 95%CI, 95% confidence interval

^a^OR and corresponding 95%CI were obtained by conditional logistic regression models with adjusting for the effects of age, education, resident area, intake of salted processed meats and dried fish, and consumption of citrus fruits and raw vegetables.

^b^P for trend was estimated excluding unknown group,

^c^excluding additionally ex-smokers group.

^d^P values for homogeneity were estimated using likelihood ratio test.

There was no significant interaction between the effects of WPT and cigarette smoking on GC risk (Table 5). The exclusively WPT smokers showed the highest GC risk.

**Table 5:** Combined effects of waterpipe tobacco smoking and cigarette smoking on gastric cancer risk.

Cigarette smoking	WPT smoking	Control		Gastric cancer		OR (95%CI)^a^
		n	%	n	%	
**Never**	Never	111	30.6	65	23.3	1.0
	Current	48	13.2	64	22.9	2.7 (1.5–4.8)
**Current**	Never	128	35.3	85	30.5	1.5 (0.9–2.4)
	Current	76	20.9	65	23.3	1.6 (0.9–2.9)

Abbreviation: OR, odds ratio; 95%CI, 95% confidence interval

^a^OR and corresponding 95%CI were obtained by conditional logistic regression models with adjusting for the effects of age, education, resident area, intake of salted processed meats and dried fish, consumption of citrus fruits and raw vegetables.

### Tumor location

Information of tumor location was able to be retrieved for only 228 (50.2%) GC cases including 41 non-antrum (18%) and 187 antrum (82%). Using the limited number of subjects, GC risk for current WPT smokers was significantly high in the antrum cases (OR = 1.7, 95%CI = 1.1–2.6) but that was not true in the non-antrum cases (OR = 1.1, 95%CI = 0.3–3.6). The association of WPT smoking with GC by tumor location after excluding cigarette smokers could not be examined because of the small number of subjects.

## Discussion

To our knowledge, this is the first case-control study to examine the association of Vietnamese WPT smoking with GC risk. The present study showed a significantly high GC risk among current WPT smokers (OR = 1.8, 95%CI = 1.3–2.4), and this association was much stronger after excluding cigarette smokers (current smokers of WPT only: OR = 2.7, 95%CI = 1.2–6.5). This might be because that the daily frequency of WPT among exclusively WPT smokers was higher than that of both WPT and cigarette smokers (the median frequency was 10 and 5.8 (Table 3), respectively). Furthermore, GC risk tended to be higher with the daily frequency, duration, and early start of WPT smoking. Although these associations were not statistically significant among exclusively WPT smokers (Table 4), trend tests gave significant results when cigarette smokers were included (P values for trend were 0.001, <0.001 and 0.003 for daily frequency, duration, and early start of WPT smoking, respectively).

Our findings are consistent with the result of hookah smoking in a previous Iranian study [7]. This Iranian cohort study reported more than three-fold higher GC risk in hookah smokers (relative risk = 3.4, 95%CI = 1.7–7.1) even after adjusting for the effects of other confounding factors, which might be because of the high-risk study population, namely *H*. *pylori*-infected subjects. Other studies have also suggested that tobacco smoking may increase the carcinogenic effect of *H*. *pylori* [29, 30]. This interaction was not examined in our study.

The Iranian study [7] also reported a significant increase of GC risk by cigarette smoking (relative risk = 3.2, 95%CI = 1.4–7.5) as reported in previous studies [24]. However, our study did not find the association between cigarette smoking and GC risk. One of the possible explanations is the relatively small number of cigarettes in our study subjects; the median of cigarette was 10 and 6 per day for current smokers of cigarette only and both WPT and cigarette smokers (Table 3), respectively. Most of the recent case-control studies have shown no increase of GC risk among smokers less than 10 cigarettes per day [24].

The present study observed no association between alcohol drinking and GC risk. The association of alcohol drinking with GC risk has not been consistent in previous epidemiological studies [1, 2, 5, 29].

Regarding the consumption of vegetables and fruits, IARC working group reported that higher intake of fruits “probably” and vegetable “possibly” reduced GC risk [27]. In our study, higher intake of citrus fruits was associated with the lower risk of GC (OR = 0.6, 95%CI = 0.4–0.8, P for trend = 0.002) but raw vegetable consumption was not related to GC risk. This finding is consistent with the result of the quantitative systematic review on citrus fruit and stomach cancer risk (OR = 0.72; 95% CI = 0.64–0.81; P value <0.0001) [31].

The smoke of Arabian-type WPT, hookah, contains a large range of carcinogenic and toxic substances as tar, nicotine, CO, PAH, aldehydes and heavy metals [32, 33], and the levels of some of them in the smoke of hookah (one-hour exposure) were equal to or higher than those in the smoke of 10 cigarettes (equivalent to 50-min exposure): tar, CO, PAHs, aldehydes, chromium, and lead [10].

Vietnamese/Chinese WPT may have lower carcinogenic effects than Arabian ones (hookah, shisha, or narghile) because of nonuse of charcoal and a very short time of one smoking session. However, a significantly high level of CO was also identified in the exhalation of Chinese WPT smokers [16] despite charcoal, a suspected main source of CO and PAHs [17], not being used. This is also true for cigarette / cigar. Although cigarette / cigar do not require charcoal, CO and PAHs level are high among smokers of these tobacco products [21]. The duration of smoking session for Vietnamese WPT is short (approximately 5 min) but the median frequency of WPT smoking per day among exclusively WPT smokers was 10 (Table 3) which is equivalent to one session of Arabian WPT smoking (45–60 min). Thus, we cannot deny a possibility that Vietnamese/Chinese WPT has similar carcinogenic effects as well as Arabian WPT. More basic examinations are necessary to estimate the levels of carcinogens in the smoke of Vietnamese/Chinese WPT.

In this study, the proportion of current smokers, either cigarette or WPT, was 54% in control patients which was similar to that in the nation-wide survey in Vietnam, 51.2% [14]. On the other hand, around 50% of smokers were WPT smokers, including smokers of both cigarette and WPT, which was higher than that in the nation-wide survey, 31%. This difference could be explained by the study area of the present study, the North Vietnam, where WPT smoking is more popular than other parts of Vietnam [14].

The present study has some limitations. First, the information on tumor location of GC was not retrieved completely. Approximately 50.1% (228) GC cases had information on tumor site, in which 82% cases were antral GC. The OR of antral GC in current WPT smokers (OR = 1.7, 95%CI = 1.1–2.6) was similar to that of all GC cases (OR = 1.8, 95%CI = 1.3–2.4), suggesting that most of GC in this study might be antral GC. Furthermore, no difference in the effect of tobacco smoking on GC risk by tumor location was reported in several case-control and cohort studies [1, 2, 5, 10].

Second, information of histological type of GC (intestinal or diffuse type) was not obtained, and we could not examine the effect modification by histological type of tumor. A hospital-based case-control study in Japan reported that habitual smoking was associated more likely with differentiated (intestinal) type of GC but the difference in the magnitudes of OR between differentiated (intestinal) and non-differentiated (diffuse) types was not significant [34]. Unlike the histological distribution of Japanese GC, in which 54% and 45% for intestinal and diffuse types, respectively, most Vietnamese GCs were intestinal type (82.7%) [35], suggesting that our findings were mainly from the results of intestinal type of GC.

In conclusion, the present study found an association of WPT smoking with GC risk among Vietnamese men. It supports the WHO’s advisory note on health effects of WPT smoking in 2015 [15].
